# IFN-I inducible *miR-3614-5p* targets ADAR1 isoforms and fine tunes innate immune activation

**DOI:** 10.3389/fimmu.2022.939907

**Published:** 2022-07-22

**Authors:** Françoise Vuillier, Zhi Li, Iain Black, Melania Cruciani, Erminia Rubino, Frédérique Michel, Sandra Pellegrini

**Affiliations:** ^1^ Cytokine Signaling Unit, Department of Immunology, Institut Pasteur, Paris, France; ^2^ Microenvironment and Immunity Unit, Institut Pasteur, Paris, France

**Keywords:** type I interferon, microRNA, ADAR1, TRIM25, innate response

## Abstract

Regulation of innate immune responses is essential for maintenance of immune homeostasis and development of an appropriate immunity against microbial infection. We show here that *miR-3614-5p*, product of the *TRIM25* host gene, is induced by type I interferon (IFN-I) in several human non-immune and immune cell types, in particular in primary myeloid cells. Studies in HeLa cells showed that *miR-3614-5p* represses both p110 and p150 ADAR1 and reduces constitutive and IFN-induced A-to-I RNA editing. In line with this, activation of innate sensors and expression of IFN-β and the pro-inflammatory IL-6 are promoted. *MiR-3614-5p* directly targets *ADAR1* transcripts by binding to one specific site in the 3’UTR. Moreover, we could show that endogenous *miR-3614-5p* is associated with Ago2 and targets *ADAR1* in IFN-stimulated cells. Overall, we propose that, by reducing ADAR1, IFN-I-induced *miR-3614-5p* contributes to lowering the activation threshold of innate sensors. Our findings provide new insights into the role of *miR-3614-5p*, placing it as a potential fine tuner of dsRNA metabolism, cell homeostasis and innate immunity.

## Introduction

MicroRNAs (miRNAs) are non-coding RNAs of ~22 nucleotides that post-transcriptionally downregulate gene expression through translational destabilization, blockage or cleavage of mRNA targets in a specific base pairing recognition manner (1). These small RNA molecules have been found involved in every biological process and are considered important regulatory elements also in the control of innate defense against invading pathogens. Numerous miRNAs have been shown to curb or promote innate immune cell activation at multiple layers by targeting pattern recognition receptors, adaptors, positive and negative signaling components, transcription factors and regulators. Thereby these tuning molecules contribute to homeostasis, production of immune cytokines and magnitude of the inflammatory response (2).

Type I interferons (IFN-I) are innate cytokines critical for the immediate host defense against viral infection. They are induced upon cell recognition of microbial products and act by up-regulating effector molecules of cell-intrinsic antiviral immunity. Moreover, they modulate functions of many immune cell populations and orchestrate innate and adaptive antiviral responses. IFN-I also mediate protective effects in other pathological contexts, such as some cancers and multiple sclerosis, but if improperly produced they can exert damaging effects, as in some bacterial infections and autoimmune diseases (3). Pathways leading to the production of IFN-I and the signaling pathway activated by IFN-I are highly regulated and can be impacted by numerous miRNAs (4). Conversely, little is known on miRNAs whose abundance can be directly modulated by IFN-I at the level of transcription or processing. Here we report on *miR-3614-5p* that we found upregulated by IFN-I in several human cell types.

Present knowledge on *miR-3614-5p* is still limited. This miRNA was identified in a few studies exploring differential miRNA expression in cancer and was ascribed both pro- and anti-tumoral effects. *MiR-3614-5p* was found to be more expressed in cervical cancer (5) and in breast cancer tissues (6) with respect to adjacent normal tissues. Conversely, *miR-3614-5p* was proposed to attenuate proliferation and invasion of non-small cell lung cancer cells by targeting the glycolysis enzyme phosphoglycerate mutase 1 (PGAM1) (7) and the WNT pathway-related NFATC2 transcription factor (8). Another study suggested a role of *miR-3614-5p* in opposing hepatocarcinoma cell proliferation and migration by targeting the transcription factor YY1 (9). The analysis of data from the Cancer Genome Atlas (TCGA) revealed lower *miR-3614-5p* expression in colorectal cancer tissues compared to adjacent normal tissue, making it a potential prognostic marker of low survival (10).

A role of *miR-3614-5p* in autoimmunity was suggested by the identification of a Crohn’s disease risk single nucleotide polymorphism possibly associated with *miR-3614-5p* (11). A reduced level of *miR-3614-5p* was correlated with inflammation in the epicardial adipose tissue of coronary artery disease patients and studies in monocytic-like THP1 cells suggested a role of *miR-3614-5p* in downmodulating inflammatory cytokine response through targeting TRAF6 (12). Recently, the presence of *miR-3614-5p* and two other miRNAs (*miR-125a-3p* and *miR-1246*) in plasma exosomal RNA from dermatomyositis patients was correlated with skeletal muscle damage (13).

No unique comprehensive picture has emerged so far on the role of *miR-3614-5p*. Interestingly, *miR-3614-5p* was scored in several genome-wide surveys of miRNAs that are modulated in response to infection. *MiR-3614-5p* was found up-regulated in human cells latently infected with Kaposi’s sarcoma-associated herpes virus (14), in human monocyte-derived DC infected with bacterial species (15), in human macrophages infected with Dengue virus (16), and in primary monocytes infected with influenza A virus or stimulated with a TLR7/8 ligand (17).

Here we have studied the expression of *miR-3614-5p* in some human cell lines and in myeloid cells. Through functional analysis in HeLa cells we demonstrate that *miR-3614-5p* is a *bona fide* IFN-I-induced miRNA that, by directly targeting the two ADAR1 isoforms, takes part in the control of RNA editing.

## Materials and methods

### Antibodies and reagents

We used antibodies against ADAR1 (Cell Signaling Technology), Ago2 (MBL, Life Science), tubulin (Merck) and IRF9 (gift from D.E. Levy, NYU School of Medicine, NY, NY). Irrelevant mouse IgG was purchased from R&D. Recombinant IFN-α2b was a gift from D. Gewert (Wellcome Foundation, Beckenham, Kent, UK). IFN-β produced by Biogen Idec (Cambridge, MA, USA) was a gift of G. Uzé. Horseradish peroxidase (HRP)-conjugated anti-rabbit IgG and anti-mouse IgG were purchased from Jackson. GM-CSF, IL-4 and M-CSF were purchased from Miltenyi Biotec.

### Isolation of monocytes and derived macrophages and dendritic cells

Peripheral blood mononuclear cells (PBMCs) and monocytes were isolated from freshly collected buffy coats as previously described (18). Freshly isolated monocytes were differentiated in macrophages or immature dendritic cells (DC). Monocytes were resuspended in complete medium RPMI 10% FCS supplemented with IL-4 (50 ng/ml) GM-CSF (100 ng/ml) for 6 days to obtain immatures monocyte-derived DC (Mo-DC) or with M-CSF (100 ng/ml) for 6 days for monocyte-derived macrophages. Medium was renewed every third day. Immature Mo-DC were analyzed for lack of CD14 and acquisition of DC-SIGN marker.

### Cell lines

HEK293T, HeLa S3, HepG2 and A549 cell lines were cultured in DMEM Glutamax (Gibco) supplemented with 10% fetal calf serum. THP1, HuT78 and Jurkat cells were cultured in RPMI Glutamax (Gibco) supplemented with 10% fetal calf serum (Pan-Biotech), non essential amino acids and sodium pyruvate.

### RNA extraction and RT-qPCR

Total RNA was extracted from Trizol using the miRNeasy mini kit (Qiagen, Germantown, MD, USA) following the manufacturer’s instructions. Quantification and purity were assessed by Nanodrop spectrophotometer (Nanodrop2000, Thermo Fisher Scientific). For measuring *ADAR1* total or *p150*, *TRIM25*, *IL-6* and *IFN-β* transcripts, total RNA was reverse-transcribed using high-capacity cDNA reverse transcription (RT) kit (Applied Biosystems, Thermo Fisher Scientific). Quantitative PCR (qPCR) assays were performed using the FastStart SYBR Green Master Mix (Roche) on QuantStudio 3 Real-Time PCR system (Applied Biosystems, Thermo Fisher Scientific). Transcript levels were normalized to *18S* levels using the equation 2^-ΔCt^. See **Table S1** for oligonucleotide sequences.

*MiRNA* expression was determined as previously described (18).

### Protein analysis

Whole-cell extracts were prepared and western blots were performed as previously described (19). Signals were detected with an enhanced chemiluminescence (ECL) detection reagent (SuperSignal^®^ WestPico Chemiluminescent Substrate, Thermo Scientific). Images were acquired with a Fuji Image Quant LAS-4000 machine. The data were analyzed by using Multi Gauge v3.2 (Fuji Image analyzing application) software. Quantified bands were normalized with respect to tubulin, as indicated in the figure legends.

### Reverse transfection of mimic or *miRNA* inhibitor

MiRIDIAN miRNA mimic and inhibitor specific for *miR-3614-5p* were purchased from Horizon Discovery Ltd. MiRIDIAN miRNA Mimic Negative Control #1 and miRIDIAN miRNA Inhibitor Negative Control #1 (Horizon Discovery Ltd) were used to assess specificity of the effect driven by the specific miRNA sequences. Mimic (50 nM) and inhibitor (100 nM) reverse transfections in HeLa S3 cells were performed as previously described (18).

### Analysis of RNA editing

The cDNA fragments containing the candidate editing sites were amplified for *AZIN1* using the primers AZIN1-Frw and AZIN1-Rev, and for *EIF2AK2* with EIF2AK2-Frw and EIF2AK2-Rev primers. PCR was performed with Phusion DNA polymerase (Thermo Scientific). A-to-I/G RNA editing was detected by sequencing the PCR products using AZIN1-seq Rev or EIF2AK2-Rev primers (**Table S1**). The editing event is given by a mixture of A+G peaks in the sequence chromatogram and measured calculating the ratio between the area of the peak corresponding to G and the sum of the areas of the double peaks A+G. Sequence analysis was performed with the software Bioedit. For the sequence of the primers used refer to **Supplementary Table 1**.

### Luciferase reporter assay

For cloning the *ADAR1* 3’UTR in the psiCHECK2 vector (Promega, Madison, WI, USA), the first 1500 nucleotides of *ADAR1* 3’UTR were amplified from HeLa S3 cDNA and cloned downstream of the *Renilla* luciferase ORF using NotI and XhoI restriction sites. The resulting plasmid (psiCHECK2-*ADAR1* 3’UTR) was mutated in the binding sites of *miR-3614-5p* (**Figure 5B**) using QuikChange XL site-directed mutagenesis kit (Aligent Technologies, Santa Clara, CA, USA) and specific primers for each site (215-219mut Frw/Rev, 589-593mut Frw/Rev, 687-691mut Frw/Rev, and 1175-1179mut Frw/Rev) (**Table S1**). New plasmids were sequenced. Mut215, mut591, mut689, and mut1175 are mutated in a single site, mut215+1175, mut591+1175 and mut689+1175 are mutated in two sites, mut591+689+1175 are mutated in three sites. For the luciferase reporter assay, HeLa S3 cells were reverse transfected with *miR-3614-5p* mimic (see above). After 24 hr, plasmid transfection was carried out using FuGENE HD (Promega) according to the manufacturer’s instructions. Three biological replicates were prepared for each condition. Cells were lysed 24 hr post-transfection and analyzed with the Dual-Luciferase^®^ Reporter Assay System (Promega) according to the manufacturer’s instructions. Firefly luciferase was measured as normalizer.

### Ago2-miRNA-co-immunoprecipitation assay

Antibody-coated protein G/protein A-agarose was prepared by incubating protein G/protein A-agarose (Calbiochem) with anti-human Argonaute2 (Ago2) antibody or irrelevant mouse IgG (R&D) overnight at 4°C. HeLa S3 cells were stimulated with IFN-α2 (100 pM) for 24 hr or left untreated and lysed in lysis buffer as previously reported (20). Lysates were cleared by centrifugation (14,000 x g) for 10 min at 4°C. Supernatants were collected and pre-cleared with protein G/protein A-agarose. Cleared lysates were then incubated with the antibody-coated protein G/protein A-agarose for 3 hr at 4°C. Bound material was washed five times with lysis buffer. Finally, a fraction (10%) was used for western blotting. The remaining fraction was digested with proteinase K and used for RNA extraction.

## Results

### *MiR-3614-5p* is induced by IFN-I and targets ADAR1

**Figure 1:**
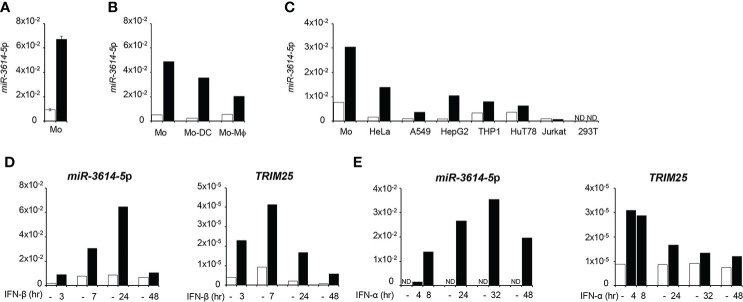
*MiR-3614-5p* is induced by type I IFN. **(A)** *MiR-3614-5p* expression in monocytes purified from two healthy donors and treated with 100pM IFN-β for 24 hr (black) or not treated (white). Results are shown as expression (2^-ΔCt^) relative to *U6* used as housekeeping gene. Values are means ± SEM. **(B)** *Mir-3614-5p* expression in monocytes from one donor and in derived macrophages (Mo-Mϕ) and dendritic cells (Mo-DC). IFN-β stimulation and results as in A). **(C)** *Mir-3614-5p* expression in different human cell lines and in primary monocytes from a single donor (Mo). Stimulation with IFN-α (100pM) for 8 hr. Data are representative of two independent experiments. ND, not detected. **(D)** *MiR-3614-5p* (left panel) and *TRIM25* (right panel) levels measured in primary monocytes stimulated with IFN-β (100pM) as indicated. Results for *TRIM25* are shown as expression (2^-ΔCt^) relative to *18S*. Data are representative of two independent experiments. **(E)** *MiR-3614-5p* (left panel) and *TRIM25* (right panel) levels measured in HeLa cells stimulated with IFN-α (100pM) for the indicated times. Results expressed as in D). Data are representative of four independent experiments.

Here we asked whether the expression of *miR-3614-5p* can be directly modulated by IFN-I. We chose myeloid cells as they are key producers and target cells of IFN-I early during immune response activation. Stem-loop RT-qPCR was performed on RNA isolated from primary monocytes, monocyte-derived dendritic cells and monocyte-derived macrophages that were stimulated or not by IFN-β (**Figures 1A, B**). *MiR-3614-5p* was found to be lowly basally expressed and induced by IFN in all subsets, most highly in undifferentiated monocytes. The level of *miR-3614-5p* was also compared in monocytes and the human cell lines HeLa, A549, HepG2, 293T, THP1 and in the CD4+ T cell lines HuT78 and Jurkat. As shown in **Figure 1C**, the miRNA was lowly expressed and induced by IFN-α to variable extent, except in 293T and Jurkat cells.

**Figure 2:**
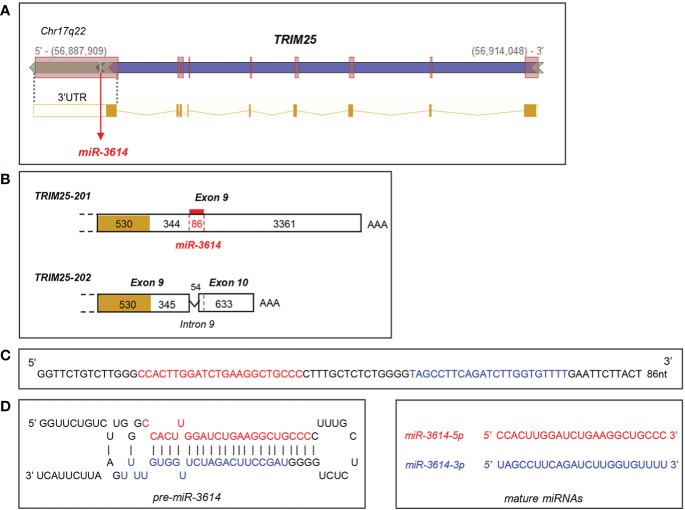
Localisation and sequence of *miR-3614*. **(A)** Genomic position of *miR-3614* sequence in the human *TRIM25* gene (from Intragenic microRNA database, miRIAD). **(B)** Localisation of *miR-3614* in the 3’UTR of *TRIM25*. Only the last exon(s) of the two protein-coding transcripts, *TRIM25-201* and *TRIM25-202*, is shown. The coding region is yellow-coloured and the 3’UTR is white-coloured. In red the 86 nt *miR-3614* sequence that overlaps an intronic splice site. **(C)** The 86 nt *miR-3614* sequence. **(D)** Secondary structure prediction of the miRNA precursor. *MiR-3614-5p* and *-3p* in red and blue, respectively.

The *miR-3614-5p* genomic sequence resides in the *TRIM25* (tripartite motif-containing protein 25) gene, a well known ISG operating in innate immune pathways (21). In particular, *pri-miR-3614* is found in the last *TRIM25* exon (exon 9) that comprises some coding sequences and a 3.79 kb-long 3’UTR (**Figure 2** **).** Given this location, we monitored in parallel the kinetics of induction by IFN-I of both *miR-3614-5p* and *TRIM25*. In primary monocytes and in HeLa cells, *TRIM25* mRNA levels peaked 4 to 8 hr after IFN addition, while *miR-3614-5p* levels peaked at later times (**Figures 1D, E**).

**Figure 3:**
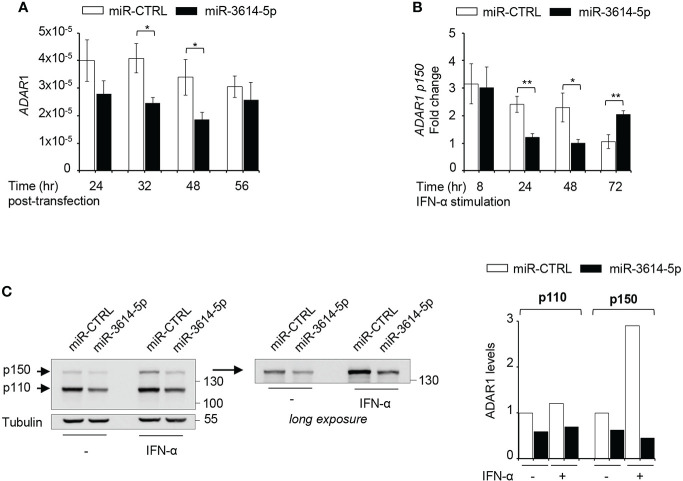
A *miR-3614-5p* mimic targets both ADAR1 isoforms. **(A)** A *miR-3614-5p* or a miR-control (miR-CTRL) mimic was retrotransfected in HeLa cells. At the indicated times post-transfection the level of total *ADAR1* mRNA was measured by qPCR. Results are shown as expression (2^-ΔCt^) relative to *18S* used as housekeeping gene. Values are means ± SEM (n=3 separate experiments). *p<0.05. **(B)** HeLa were retrotransfected as in A) with *miR-3614-5p* or a miR-control (miR-CTRL) and 48 hr later, IFN-α (100pM) was added. Relative expression of *p150 ADAR1* was measured at the indicated times after stimulation. The fold change represents the ratio of IFN-stimulated cells relative to unstimulated cells. Values are means ± SEM (n=3), *p<0.05, **p<0.01. **(C)** HeLa cells were retrotransfected as in A). Twenty-four hr later cells were stimulated with IFN-α (100pM) for 2 days and then processed for western blot analysis. Tubulin was used as loading control. Right panel, the membrane was covered below the 130 kDa marker and exposed for longer time to better visualize the p150 ADAR1 isoform. Protein bands were quantified and normalized to tubulin. For each isoform results are expressed as ratio of *miR-3614-5p-*transfected cells relative to miR-CTRL-transfected cells. The value in untreated *miR-CTRL-*transfected cells set to 1. The data are representative of three independent experiments.

We used four prediction algorithms to identify candidate gene targets of *miR-3614-5p* and one of the best scored hit was *ADAR1*, member of the adenosine deaminase acting on RNA (ADAR) family and key RNA editing enzyme preventing activation of nucleic acid sensors by self-dsRNAs (22). The *ADAR1* gene specifies two transcripts generated through alternative promoters and splicing of exon 1. One transcript is constitutively expressed and encodes the ubiquitous 110-kDa protein isoform (p110) predominantly nuclear, while the other transcript is induced by IFN-I and encodes a 150-kDa protein isoform (p150) with a unique N-ter domain allowing nuclear-cytoplasmic shuttling (23). We sought to analyze the impact of *miR-3614-5p* on *ADAR1* RNA and protein in HeLa cells. Expression of a mimic of *miR-3614-5p* caused a reduction of *ADAR1* transcripts measured by RT-qPCR (**Figure 3A**). In cells treated with IFN-α, the *miR-3614-5p* mimic led to a substantial reduction of the inducible *ADAR1 p150* transcript as well (**Figure 3B**). Of note, at 72 hr post-stimulation the effect of the mimic on *ADAR1* had waned, possibly due to reduced editing and consequent accumulation of unedited dsRNA with production of IFN-β and induction of ADAR1 p150 (see next section). The impact of *miR-3614-5p* at the protein level was studied by western blot. In non-stimulated cells, a reduction of nearly 50% of both the constitutive p110 and the weakly expressed p150 isoform was observed. In IFN-α stimulated cells, the induced p150 isoform was also considerably reduced (**Figure 3C**).

### *MiR-3614-5p* lowers dsRNA editing

**Figure 4:**
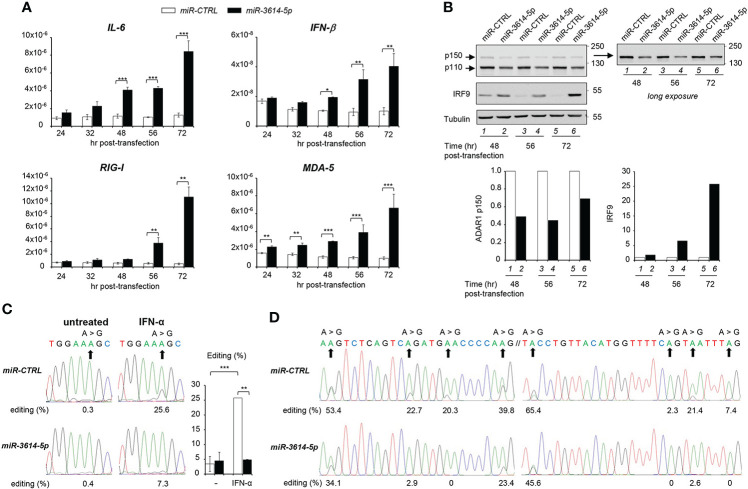
*MiR-3614-5p* impacts innate cytokine expression and RNA editing. **(A)** HeLa cells were retrotransfected with the *mir-3614-5p* or the miR-control (miR-CTRL) as mimics. The relative expression of *IL-6*, *IFN-β*, *RIG-I* and *MDA-5* was measured at different times after retrotransfection, as indicated. Values are means ± SEM (n=3 separate experiments); *p<0.05; **p<0.01; ***p<0.001. **(B)** Cells retrotransfected as in A) were lysed for western blot analysis at different times post-transfection. Lysates were immunoblotted as indicated. Tubulin was used as loading control. Right panel, the membrane was covered below the 130 kDa marker and exposed for longer time to better visualize the p150 ADAR1 isoform. Protein bands were quantified and normalized to tubulin. Results are expressed as ratios of *miR-3614-5p-*transfected cells (black) relative to miR-CTRL-transfected cells (white). Values in miR-CTRL-transfected cells were set to 1. These data are representative of two experiments. **(C)** HeLa cells were retrotransfected with the *mir-3614-5p* or the miR-control (miR-CTRL) as mimics. Two days later cells were left untreated or treated with IFN-α (100pM) for an additional 72 hr. RNA was extracted and editing was determined, as described in Materials and Methods. The percentage of editing was measured after sequencing the *AZIN1* PCR products. Percentages of editing in *AZIN1* transcripts are shown below the chromatograms of a representative experiment. Right histogram, three independent experiments were quantified. Values are means ± SEM; **p<0.01; ***p<0.001. **(D)** HeLa cells were retrotransfected with *mir-3614-5p* or miR-control (miR-CTRL) as mimics and left in culture for 56 hr. RNA was extracted and editing was determined, as described in Materials and Methods. The percentage of editing was measured after sequencing the *EIF2AK2* PCR products. Two regions of an editing box located in 3’UTR of *EIF2AK2* transcripts are represented. The two sequences have their genomic counterpart localized at chr2: 37,328,008-37,328,100 (24). Their percentages of editing are shown below the chromatograms. The data are representative of two independent experiments.

The above data showed that *miR-3614-5p* is an IFN-inducible miRNA that is able to target constitutive and inducible ADAR1. Given the key function of ADAR1 in adenosine to inosine (A-to-I) editing self and viral dsRNA, we reasoned that the *miR-3614-5p*-mediated decline in the level of steady-state ADAR1 could cause an accumulation of unedited endogenous dsRNAs, leading to aberrant activation of key sensors, like (RIG-I)-like receptors, and induction of IFN-β and pro-inflammatory cytokines. To test this possibility, we compared the level of *IFN-β* and *IL-6* mRNAs in control and *miR-3614-5p*-transfected cells. Indeed, both cytokine transcripts were up-regulated in *miR-3614-5p*-transfected cells at late times (**Figure 4A**). Moreover, the transcript level of the sensors RIG-I and MDA5 - themselves ISGs - was also augmented (**Figure 4A**). Western blot analysis of cell lysates confirmed a 50% reduction of ADAR1 in *miR-3614-5p*-transfected cells with a substantial increase in the amount of the transcription factor IRF9, an ISG product (**Figure 4B**).

To demonstrate that *miR-3614-5p* impinges on ADAR1-mediated editing, we measured editing of antizyme inhibitor 1 RNA (*AZIN1*), a well known ADAR1 substrate that is commonly used as an editing activity read-out (25). In the absence of IFN, *AZIN1* editing was barely detectable in control unstimulated cells, so that the effect of *miR-3614-5p* could not be evaluated in baseline condition. Conversely, after IFN stimulation the level of *AZIN1* editing was increased from 0.3% to 25.6% in control cells. However, in *miR-3614-5p*-transfected cells the increase was much less, from 0.4% to 7.3% (**Figure 4C**).

Having shown that *mir-3614-5p* impacts editing in IFN stimulated conditions, we asked whether *miR-3614-5p* could affect constitutive editing. Zhu et al. (24) reported on three clusters of constitutive A-to-I editing sites that are located in the 3’UTR of *EIF2AK2* (*PKR*) and were named editing boxes. Working with HeLa cells, the authors validated these sites as being edited by ADAR1 and quantified the editing ratios for each of them. In line with this, we tested whether *miR-3614-5p* could reduce the level of editing at these same sites. We measured A-to-I editing in two *EIF2AK2* regions 56 hr after retrotransfection of control or *miR-3614-5p* mimic **(** **Figure 4D** **)**. The percentages of editing in control samples were remarkably similar to those reported in (24). Importantly, we found that the expression of *miR-3614-5p* decreased editing and this whatever the level of editing (high, intermediate or low) was in the control. Thus, by targeting *ADAR1, miR-3614-5p* can impact editing in resting and in IFN-stimulated conditions. Altogether, these data suggest that *miR-3614-5p* is involved in RNA metabolism, thus contributing to regulation of the innate immune response.

### *MiR-3614-5p* directly targets the 3’UTR of *ADAR1*

**Figure 5:**
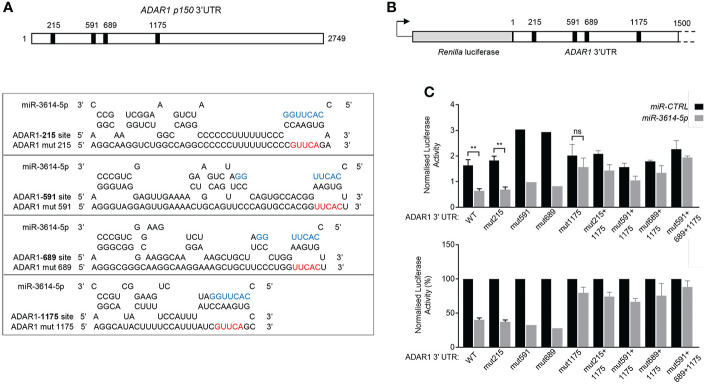
*MiR-3614-5p* targets *ADAR1* 3’UTR through one major binding site. **(A)** Schematic diagram of the 3’UTR of the *p150 ADAR1* transcript. Black boxes indicate the location of the four predicted binding sites of *miR-3614-5p*. Site 215 matches to *miR-3614-5p* positions 2-8 of the seed sequence with an A opposite to position 1. Site 1175 matches to *miR-3614-5p* positions 2-10. Site 591 and site 689 are two non-canonical 5-mer binding sites. The RNAhybrid miRNA target prediction tool was used (https://bibiserv.cebitec.uni-bielefeld.de/rnahybrid). Mutant *ADAR1* target sites (mut215, mut591, mut689, mut1175) were generated by mutating 5nt complementary to the 5’ seed region of *miR-3614-5p*. The seed sequence is in blue. The mutated nucleotides are in red. **(B)** Schematic diagram of the 1.5 kb *ADAR1* 3’UTR sequence containing the four predicted *miR-3614-5p* binding sites (black squares) cloned downstream of *Renilla* luciferase in the psiCHECK-2 vector. **(C)** Luciferase assay to assess direct interaction of *miR-3614-5p* with the *ADAR1* 3’UTR. HeLa cells were retrotransfected with the *miR-3614-5p* mimic or the non-targeting control mimic (50nM) and the next day transfected with the dual luciferase reporter psiCHECK-2 carrying the *ADAR1* 3’UTR, as in **(B)**. Twenty four hr later *Renilla* luciferase activity was measured and normalized to the internal firefly luciferase control (top panel). WT and mut1175 data: mean of four experiments each in triplicate. Mut215 data: mean of 3 experiments each in triplicate. Mut591, mut689 data: one experiment in triplicate. Data on combined mutations: mean of two experiments each in triplicate. Statistics is available only for results with 3 independent experiments. Data represented as mean ± SEM. *p<0.05, **p<0.01, ns = not significant. Normalised luciferase activities (top panel) are represented as percentage of the control mimic (bottom panel). Data are represented as mean ± SEM.

Next we sought to validate a direct impact of *miR-3614-5p* on *ADAR1* 3’UTR. The two *ADAR1* transcripts - encoding p110 and p150 isoforms - possess an identical 3’UTR, except for 10 additional nucleotides at the very 3’ end of the p150-encoding transcript. The whole 3’UTR was scrutinized using miRNA target prediction algorithms. Canonical miRNA targeting involves nucleotides 2-7 at the 5’ end of the miRNA. This “seed” sequence pairs with complementary nucleotides in the 3’UTR of mRNA (1). Complementarity with the 3’end of the miRNA can compensate for imperfect matches within the seed sequence. We scored four candidate *miR-3614-5p* target sites, all located in the 5’ half of the 3’UTR. Each site was named according to the position of the seed-pairing nucleotide. According to Agarwal et al. (26), site 215 and site 1175 are canonical sites, while site 591 and site 689 are non canonical 5-mer sites (**Figure 5A** **)**.

To study the direct and site-specific activity of *miR-3614-5p* we used a reporter assay. A 1.5 kb-long fragment of the 3’UTR encompassing the four sites was subcloned downstream of *Renilla* luciferase in the psiCHECK-2 reporter plasmid (**Figure 5B**). HeLa cells were first retrotransfected with the *miR-3614-5p* mimic or the control mimic and then transfected with the reporter plasmid. Luciferase activity was measured 24 hr later. As shown in **Figure 5C**, the *miR-3614-5p* mimic caused nearly 60% reduction of luciferase activity.

To identify which site(s) in the 3’UTR was targeted by *miR-3614-5p*, we introduced mutations in each of the four sites (**Figure 5A**). Mutation of site 215, site 591 or site 689 had minimal effects, while mutation of site 1175 reduced the effect of *miR-3614-5p* (**Figure 5C**). Combining the mutation of sites 215, 591 or 689 with that of site 1175 did not further reduce the effect of *miR-3614-5p* compared to mutation of site 1175 alone, providing further evidence that the predicted sites 215, 591 and 689 are not targeted by *miR-3614-5p* (**Figure 5C**). In conclusion, these data demonstrate that *miR-3614-5p* is able to down-modulate expression of a reporter gene containing the *ADAR1* 3’UTR and that this occurs through direct binding of the miRNA to site 1175.

### Endogenous *miR-3614-5p* is recruited to RISC and can repress *ADAR1* transcripts

**Figure 6:**
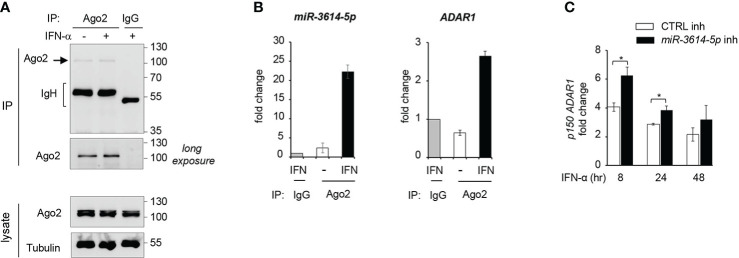
Endogenous *miR-3614-5p* is associated with Ago-2 and targets *ADAR1* in IFN-stimulated cells. **(A)** HeLa cells were stimulated with IFN-α (100pM) for 24 hr and processed for Argonaute2-miRNA-co-immunoprecipitation. Endogenous Argonaute2 (Ago2) was immunoprecipitated (IP) from whole cell lysates with anti-Ago2 or an IgG control. Samples were analysed by western blot as indicated. **(B)** *miR-3614-5p* (left panel) or *ADAR1* mRNA (right panel) was measured by qPCR in the Ago2 immunoprecipitated fraction relative to the IgG fraction, shown in A). The Ct values of *miR3614-5p* or *ADAR1* in the IgG immunoprecipitate were substracted from the Ct values in Ago2 immunoprecipitates (IFN-treated or untreated) to generate ΔCts. The results shown are expressed as fold change and represent the expression (2^-ΔCt^) relative to control IgG set at 1. Data are mean +/- SEM of two independent experiments. **(C)** HeLa cells were transfected with the *miR-3614-5p* inhibitor (*miR-3614-5p* inh) or the control inhibitor (CTRL inh). Two days later, IFN-α (100pM) was added. Relative expression of *p150 ADAR1* was measured at different times after stimulation as indicated. The fold change represents the ratio of IFN-treated cells relative to non-treated cells. Values are means ± SEM (n=3 separate experiments). *p < 0.05.

Having validated the effect of *miR-3614-5p* expressed as mimic on ADAR1, we turned to the endogenous miRNA. Argonaute2 (Ago2) is an essential component of RISC (RNA-induced silencing complex), which helps binding of miRNA to the target-site on mRNA. Hence the Ago2-associated miRNA represents the functional fraction involved in mRNA down-regulation (1). Here, we aimed to investigate the association of *miR-3614-5p* with RISC, using an Argonaute2-miRNA-co-immunoprecipitation assay (see Materials and Methods). We measured levels of *miR-3614-5p* and *ADAR1* mRNA present in Ago2 immunoprecipitates from untreated and IFN-treated HeLa cells (**Figures 6A, B**). We could show that after IFN stimulation *miR-3614-5p* was particularly enriched in Ago2 antibody-coated beads with respect to control IgG beads. In addition, we detected the presence of *ADAR1* mRNA in the Ago2 immunoprecipitated material, further supporting the binding of *miR-3614-5p* to *ADAR1* (**Figure 6B**).

Next, we aimed to deplete endogenous *miR-3614-5p* from IFN-treated HeLa cells and measure the effect on *ADAR p150* mRNA. For this, cells were retrotransfected with a *miR-3614-5p* inhibitor or a control inhibitor and then left untreated or treated with IFN for different times. As shown in **Figure 6C**, in cells treated with IFN-α the *miR-3614-5p* inhibitor led to an increase of the *ADAR1 p150* transcript. The above results on the endogenous *miR-3614-5p* reinforce our conclusion that *ADAR1* is a *bona fide* target of this IFN-induced miRNA.

## Discussion

In this study we show that the primate-specific *miR-3614-5p* is induced by IFN-I in several human immune and non-immune cell types, in particular primary myeloid cells, which are known to be central in the initiation of an immune response. Using overexpression and inhibition approaches in HeLa cells, we demonstrate that one functional target of *miR-3614-5p* is the enzyme ADAR1. We identified a major binding site in the 3’UTR of *ADAR1* transcripts through which *miR-3614-5p* plays its regulatory role and showed that, upon IFN stimulation, endogenous *miR-3614-5p* and *ADAR1* are both recruited by the RISC component Ago2.

*MiR-3614-5p* was previously identified in cultured macrophages infected with Dengue virus (16). The authors noticed that *miR-3614-5p* was upregulated not only in infected cells but also in the non-infected cells of the culture, which suggests the action of a soluble mediator, *i.e.* IFN-I, released by infected cells. With high throughput sequencing technologies, the knowledge on miRNAs that are directly modulated by IFN-I in specific cell types/tissues and physio-pathological contexts has increased. Indeed, Rodriguez-Gala et al. (27) searched for IFN-regulated miRNAs in human primary CD4+ T cells and identified 57 miRNAs that appear weakly modulated by IFN, 24 being upregulated and 33 repressed. *MiR-3614-5p* was highlighted as one of the most upregulated miRNA (8 fold after 24 hr), rising from a very low threshold level. Interestingly, *miR-3614-5p* was found to be upregulated by IFN-β also in human skeletal muscle myoblasts (13).

The *miR-3614* gene has some interesting features. The 86 nt-sequence is found only in primates (**Supplementary Figure 1**) and is likely the product of recent evolution. This sequence gives rise to mature *miR-3614-5p* and *-3p*, with -*5p* being predominant, and resides within the *TRIM25* host gene, which provides expression constraint. *TRIM25* and *miR-3614-5p* transcripts are both up-regulated by IFN-I through the IFN-responsive *TRIM25* promoter (28). However, their accumulation profiles differ. In primary monocytes and HeLa cells *TRIM25* mRNA peaked 4 to 8 hr after IFN addition, while *miR-3614-5p* peaked at later times (**Figures 1D, E**). The unique position of the pri-miRNA may account for the different accumulation profile. As schematized in **Figure 2**, *pri-miR-3614* resides in the 3.79 kb-long 3’UTR of the abundant *TRIM25-201* transcript. Interestingly, *miR-3614* belongs to the class of SO-miRNAs (splice site overlapping miRNA), since the *miR-3614* extends over a splice site [see **Table 1** in (29)]. Splicing of intronic sequences at this site generates an alternative protein-coding transcript (*TRIM25-202*) that contains a shorter 3’UTR of 0.9 kb and is expressed at very low level. Given the unusual location of *miR-3614*, *i.e.* overlapping a splice site in the *TRIM25* 3’UTR, the relative amount of *TRIM25* transcripts and *pri-miR-3614* produced may be governed by a mechanism of competition between splicing and miRNA processing machineries (30, 31). Indeed, the depletion of the splicing factor SF3b1 in HeLa cells was found to increase the level of several mature SO-miRNAs including *miR-3614-3p* (29). To try to uncover a potential cross-regulation of *TRIM25* and *miR-3614-5p*, we analyzed the effect of depleting SF3b1 on the abundance of both RNAs. Given the low level of endogenous *miR-3614-5p* in HeLa cells, we used a human TRIM25 expression plasmid (pSPORT6-TRIM25) that retains a portion of the 3’UTR (clone ID: 4419084, Open Biosytems). When transfected, this plasmid transcribes *TRIM25* as well as *miR-3614*. In cells depleted of SF3b1 we did find an increase of *miR-3614-5p* and *-3p* as reported in (29), but we did not observe a change in the relative abundance of *TRIM25-201* and *-202* isoforms (data not shown). Hence, further work is needed to address mechanistic questions on the biogenesis of *miR-3614-5p*. Moreover, the expression levels of *TRIM25* isoforms and *miR-3614-5p* will have to be assessed in different tissues/cell types and in different contexts.

TRIM25 is a RING-type E3 ligase that catalyzes ubiquitination of numerous proteins in multiple RNA-dependent pathways (21). TRIM25 plays a crucial role in the RIG-I antiviral response pathway (32), though other E3 ligases such as RIPLET can activate RIG-I (33). TRIM25 is also involved in the activation of other antiviral sensors, such as zinc finger antiviral protein or ZAP (34). Of note TRIM25 can conjugate not only ubiquitin but also ISG15, this latter being highly induced in IFN-primed cells, and TRIM25 auto-ISGylation was shown to downregulate its activity (35). TRIM25 is also known as EFP (estrogen-responsive finger protein) for its capacity to respond to estrogens *via* an enhancer element in the 3’UTR (36). TRIM25 constitutes a key regulator of metastatic gene signatures in breast cancer (37) and elevated *TRIM25* mRNA is associated with poor prognosis in breast cancer and several other cancers (38). Since, as we propose here, *miR-3614-5p*, a product of *TRIM25*, fine tunes immune response, it will be interesting to study *miR-3614-5p* expression and function in these cancer contexts.

*MiR-3614-5p* is lowly expressed at baseline and modestly induced by IFN-I, at least in the cell types we and others have studied. This feature does not suggest direct targeting of abundant viral transcripts, but rather points to a role of *miR-3614-5p* in fine-tuning host genes. We show here that the two *ADAR1* transcript isoforms, which share the 3’UTR, are directly targeted by *miR-3614-5p*, in accordance with previous work (16). The downregulation of ADAR1 by specific miRNAs has been previously reported. *MiR-1*, involved in myogenesis regulation (39, 40), *miR-143* (34), *miR-17-5p* and *miR-432* (41) were found to directly bind to *ADAR1* 3’UTR. The genes encoding *miR-17-5p* and *miR-432* are frequently amplified in melanomas and their overexpression is coupled with reduced ADAR1 expression that has been associated with aggressive features of metastatic melanomas. Of note, the highly conserved *miR-1* was shown to be induced by IFN-β in human hepatoma HuH7 cells, murine primary hepatocytes (42), and PBMC (43). Hence, it is possible that in certain contexts IFN-induced *miR-1* and *miR-3614-5p* cooperate to reduce *ADAR1* level.

ADAR1 is a dsRNA-specific deaminase that takes part in the discrimination of self *vs* non-self RNAs. Its dsRNA editing activity (conversion of adenosine to inosine) is most often exerted on paired *Alu* elements present in primate self RNAs. In any given cell type and in normal condition, the baseline level of ADAR1 sets the threshold for innate immune activation, as this enzyme restrains aberrant activation of dsRNA sensors by endogenous RNA, preventing production of IFN-I (44). Partial loss-of-function mutations in ADAR1 cause an autoinflammatory Aicardi-Goutières syndrome-related interferonopathy in humans (45). In infectious contexts, ADAR1 has been regarded as proviral for its ability to edit dsRNA and to competitively bind dsRNA, restraining activation of immune sensors such as MDA-5 and PKR, a potent inhibitor of translation (46). However, for some viruses ADAR1-mediated editing can be regarded as having an antiviral effect (47).

In cancer the influence of ADAR1 is complex. It has been shown to have mostly pro-oncogenic effects exerted through diverse and context-specific mechanisms (48). Several cancer cell lines rely on ADAR1 for survival (49). High *AZIN* editing in colorectal cancer was correlated with disease severity (50). However, in melanoma, loss of ADAR1 was reported to promote metastatic growth due to deregulation of several miRNAs (41). Interestingly, loss of ADAR1 was shown to sensitize tumor cells to immunotherapy and to contribute to overcome resistance to checkpoint blockade at least in part through increased IFN-I production (51).

**Figure 7:**
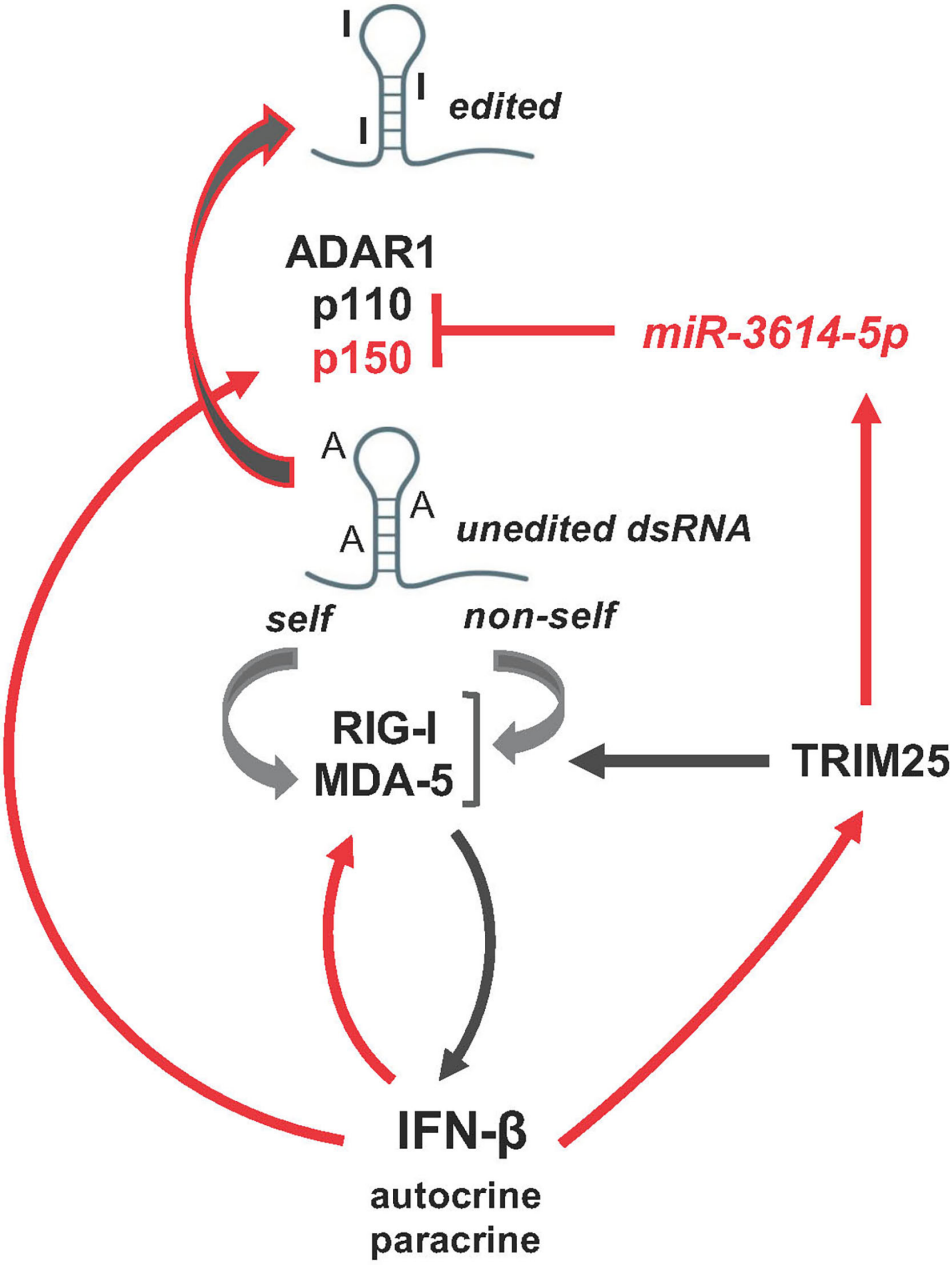
Schematic representation of *miR-3614-5p* involvement in the network of activation of the dsRNA sensors RIG-I and MDA-5 mediating induction of IFN-I and inflammatory response. Of these intricate pathways only the few actors studied here are depicted. Red arrows, transcriptional gene induction by IFN-I, which acts in an autocrine and paracrine fashion.

Our functional studies in HeLa cells indicate that IFN-induced *miR-3614-5p* impacts on the level of ADAR1 proteins and dsRNA editing, eventually promoting activation of innate immune signaling with upregulation of IFN-β and IL-6 (scheme in **Figure 7**). In this view, *miR-3614-5p* upregulation can be interpreted as reducing the threshold of activation of innate sensors, such as RIG-I and MDA-5, in an IFN-autocrine/paracrine dependent manner, thereby facilitating an efficient antiviral response.

Having shown that ADAR1 is a direct target of *miR-3614-5p* adds up to the multiple regulatory mechanisms that have been shown to modulate ADAR1 levels and functions (52) and opens up questions about the level of *miR-3614-5p* in contexts where ADAR1 was shown to exert proviral, antiviral, oncogenic or tumor suppressor roles. Obviously, the investigation of the single *miR-3614-5p*-*ADAR1* interaction has its limitation, since other mRNAs are expected to be targeted by this miRNA in a cell-type and context-specific manner. Since *miR-3614-5p* has most likely emerged in primates, research in mouse models cannot be carried out, but high-throughput technologies and computational approaches should help to fully appreciate the complexity of gene regulation by *miR-3614-5p* and its possible involvement in immune dysregulation in disease.

## Data Availability Statement

The original contributions presented in the study are included in the article/**supplementary material** Further inquiries can be directed to the corresponding author.

## Author Contributions

FV, MC, ER, ZL, FM and SP designed the study. FV, MC, ZL and IB performed experiments. SP and FV wrote the manuscript. All authors read and provided input on the manuscript and approved the final version. All authors contributed to the article and approved the submitted version.

## Funding

This work was funded by the Fondation pour la Recherche Médicale (Equipe FRM DEQ20170336741) and institutional funds from Institut Pasteur and Institut national de la santé et de la recherche médicale (Inserm). ZL was supported by Centre national de la recherche scientifique (CNRS). ER was supported by FRM. MC was supported by the FRM grant above. IB was supported by the Erasmus+ EU programme (University of Glasgow).

## Conflict of Interest

The authors declare that the research was conducted in the absence of any commercial or financial relationships.

## Publisher’s Note

All claims expressed in this article are solely those of the authors and do not necessarily represent those of their affiliated organizations, or those of the publisher, the editors and the reviewers. Any product that may be evaluated in this article, or claim that may be made by its manufacturer, is not guaranteed or endorsed by the publisher.

## Supplementary Material

Supplementary Figure 1 Comparative analysis of *miR-3614* in human and non-human primates. **(A)** Phylogenetic tree and **(B)** sequence alignment of *miR-3614* (human chr17:56891270-56891355) in 24 primates. https://www.ensembl.org/Homo_sapiens/Location/Compara_Alignments?db=core;g=ENSG00000284542;r=17:56891270-56891355;t=ENST00000581261;time=1649078338264.264.
