# Supplementary figures and images for: IFN-I inducible miR-3614-5p targets ADAR1 isoforms and fine tunes innate immune activation
Source: Front Immunol. 2022 Jul 22;13:939907. doi: 10.3389/fimmu.2022.939907 (PMC9354889; doi:10.3389/fimmu.2022.939907)

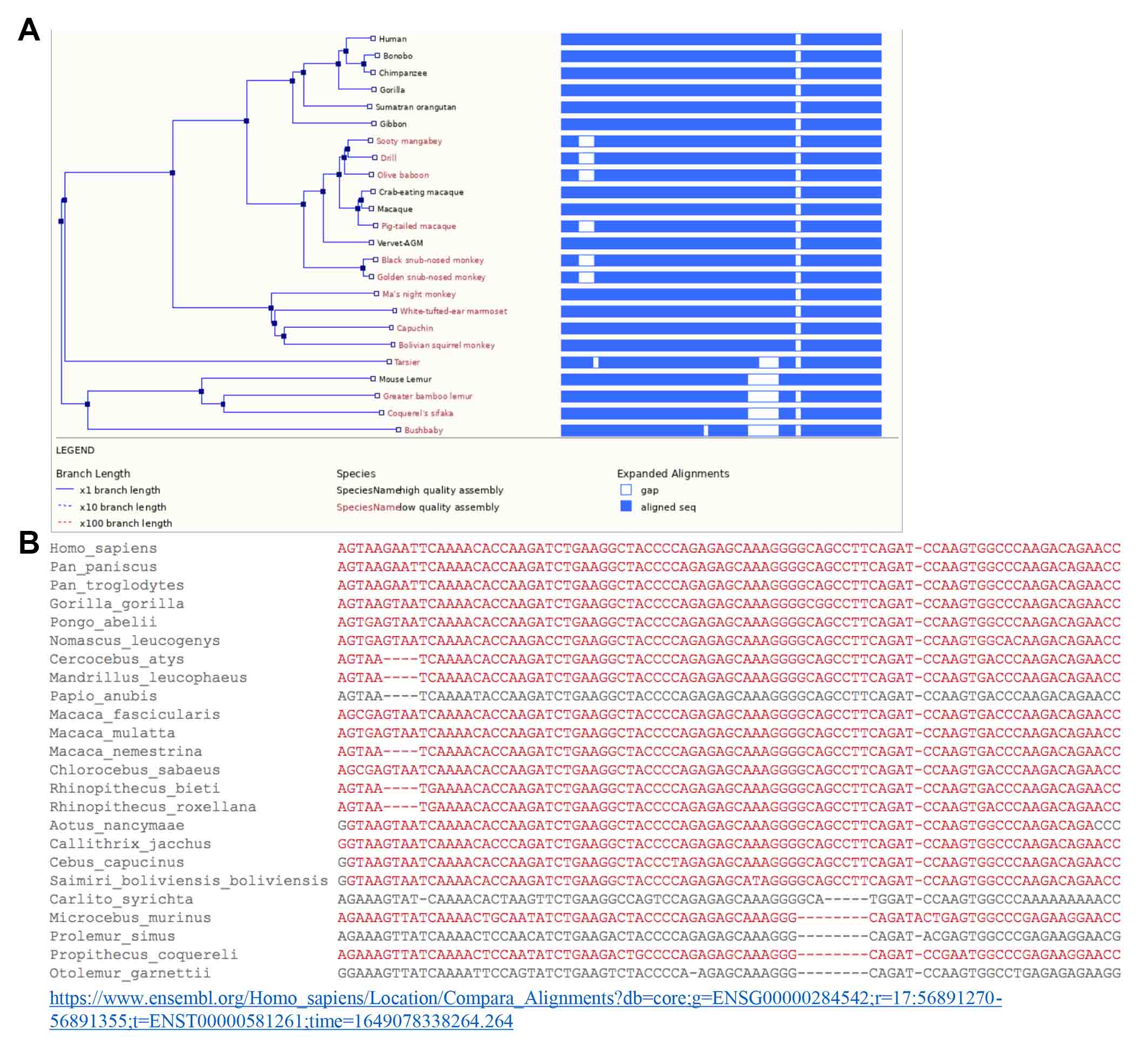

Supplement: Supplementary Figure 1 — Comparative analysis of miR-3614 in human and non-human primates. (A) Phylogenetic tree and (B) sequence alignment of miR-3614 (human chr17:56891270-56891355) in 24 primates. https://www.ensembl.org/Homo_sapiens/Location/Compara_Alignments?db=core;g=ENSG00000284542;r=17:56891270-56891355;t=ENST00000581261;time=1649078338264.264. [file Image_1.jpeg]
