# Supplementary material for: IFN-I inducible miR-3614-5p targets ADAR1 isoforms and fine tunes innate immune activation
Source: Front Immunol. 2022 Jul 22;13:939907. doi: 10.3389/fimmu.2022.939907 (PMC9354889; doi:10.3389/fimmu.2022.939907)
Supplement: Supplementary file 2 [file Table_1.docx]

| **Table S1. List of primers used** | |
| --- | --- |
| **Primers** | **Sequence 5' to 3'** |
| 18S-Frw | cat ggc cgt tct tag ttg gt |
| 18S-Rev | cgc tga gcc agt cag tgt ag |
| total ADAR1-Frw | cgc cct ctt tga caa gtc ct |
| total ADAR1-Rev | atg gta cgg agt ctc tcc cc |
| p150 ADAR1-Frw | cgg gca atg cct cgc |
| p150 ADAR1-Rev | aat gga tgg gtg tag tat ccg c |
| AZIN1-Frw | tgt tta caa gca gcc ttt gg |
| AZIN1-Rev | ttc cag cat ctt gca tct ca |
| AZIN-seq-Rev | tca tgg aaa gaa tct gct ccc |
| EIF2AK2-Frw | tgg atg tgg gga tta agg aa |
| EIF2AK2-Rev | cct caa gct cac tgt cac ca |
| IFNβ-Frw | gtc tcc tcc aaa ttg ctc tc |
| IFNβ-Rev | aca gga gct tct gac act ga |
| IL-6-Frw | aat gag gag act tgc ctg gtg |
| IL-6-Rev | tgg gtc agg ggt ggt tat tg |
| MDA5-Frw | aga agg agg tct ggg gca tgg a |
| MDA5-Rev | ctc cgg gga tgc tct tgc tgc |
| RIG I-Frw | gca tga cca ccg agc agc ga |
| RIG I-Rev | agc cac gga acc agc ctt cct |
| TRIM25-Frw | gcg tcc aca cac aaa tcc ac |
| TRIM25-Rev | agt tcg gat gtg agc tgg tg |
| ADAR1-3'UTR 215-219mut Frw | cca ggc ccc cct ttt ttc ccc gtt cag aag agg cag aaa cct aag aa |
| ADAR1-3'UTR 215-219mut Rev | ttc tta ggt ttc tgc ctc ttc tga acg ggg aaa aaa ggg ggg cct gg |
| ADAR1-3'UTR 589-593mut Frw | ctg cag ttc cca gtg cca cgg ttc act gat tgg agc ctg cag ata at |
| ADAR1-3'UTR 589-593mut Rev | att atc tgc agg ctc caa tca gtg aac cgt ggc act ggg aac tgc ag |
| ADAR1-3'UTR 687-691mut Frw | caa gga aag ctg ctt ccc tgg ttc act atc act ttc tcc ggc agc tg |
| ADAR1-3'UTR 687-691mut Rev | cag ctg ccg gag aaa gtg ata gtg aac cag gga agc agc ttt cct tg |
| ADAR1-3'UTR 1175-1179mut Frw | att taa aat tca ggc ata ctt ttc cat tta tcg ttc agc ttt cat ttt tcc aga tgg ctt cag aag tag |
| ADAR1-3'UTR 1175-1179mut Rev | cta ctt ctg aag cca tct gga aaa atg aaa gct gaa cga taa atg gaa aag tat gcc tga att tta aat |
